# Altered tear fluid protein expression in persons with mild Alzheimer's disease in proteins involved in oxidative stress, protein synthesis, and energy metabolism

**DOI:** 10.1177/13872877251326868

**Published:** 2025-04-04

**Authors:** Virve Kärkkäinen, Toni Saari, Sanna Hannonen, Minna Rusanen, Juha-Matti Lehtola, Hannu Uusitalo, Ville Leinonen, Bernd Thiede, Kai Kaarniranta, Anne M Koivisto, Tor P Utheim

**Affiliations:** 1 NeuroCenter, Neurology, Kuopio University Hospital, Kuopio, Finland; 2 NeuroCenter, Neurosurgery, Kuopio University Hospital, Kuopio, Finland; 3 Neurosurgery, Institute of Clinical Medicine, School of Medicine, University of Eastern Finland, Kuopio, Finland; 4 Institute for Molecular Medicine Finland (FIMM), HiLIFE, University of Helsinki, Helsinki, Finland; 5 Neurology, Institute of Clinical Medicine, School of Medicine, University of Eastern Finland, Kuopio, Finland; 6 Ceriatric Center, Wellbeing Services Country of North Karelia, Joensuu, Finland; 7 Eye and Vision Research, Faculty of Medicine and Health Technology, Tampere University, Tampere, Finland; 8 Department of Biosciences, University of Oslo, Oslo, Norway; 9 Department of Ophthalmology, Institute of Clinical Medicine, School of Medicine, University of Eastern Finland and Kuopio University Hospital, Kuopio, Finland; 10 Department of Molecular Genetics, University of Lodz, Lodz, Poland; 11 Department of Geriatrics, Helsinki University Hospital and Department of Neurosciences, University of Helsinki, Helsinki, Finland; 12 Department of Ophthalmology, University of Oslo, Oslo, Norway; 13 Department of Medical Biochemistry, Oslo University Hospital, Oslo, Norway

**Keywords:** Alzheimer's disease, oxidative stress, pathophysiology, proteomics, reactive oxidative species, tear fluid

## Abstract

**Background:**

Tear fluid (TF) is a protein-rich solution that reflects pathophysiological changes in Alzheimer's disease (AD).

**Objective:**

In this study, we examined whether TF proteins were differently expressed in persons with mild AD dementia compared to cognitively healthy controls (CO).

**Methods:**

We analyzed data from 53 study participants including 34 CO (mean age, 71 years; Mini-Mental State Examination [MMSE] score, 28.9 ± 1.4), and 19 patients with AD (Clinical Dementia Rating, 0.5–1; mean age, 72 years; MMSE score, 23.8 ± 2.8). All participants underwent cognitive testing, as well as neurological and ophthalmological examinations. TF was collected using Schirmer strips, and TF protein content was evaluated using mass spectrometry-based proteomics and label-free quantification.

**Results:**

We found that 16 proteins exhibited significantly upregulated expression in the AD group compared to the CO group (*p *≤ 0.05). These proteins were NP1L4, BBOX1, CYTC, RNAS4, PCD, RNT2, AL1A3, SYSC, TPIS, CLH1, PGAM1, EIF3L, 5NTC, HNRNPA2B1, PYGL, and ERO1α. No proteins were significantly downregulated in the AD group compared to the CO group.

**Conclusions:**

Our results support the hypothesis that TF is a potential source of biomarkers for AD. Part of those proteins with altered expression have previously linked to increased oxidative stress, changed protein synthesis, and disturbed regulation of energy metabolism related to AD or neurodegenerative disease. The present results indicate the value of continued investigation of TF proteins in AD.

## Introduction

Alzheimer's disease (AD) is the most common progressive neurodegenerative disease worldwide. AD-related pathophysiological changes in the brain typically start to develop several years before the person recognizes the first memory deficits.^1^ By the time AD is diagnosed, typical AD symptoms are often already present and, importantly, pathological changes in the brain are widespread.^2^ It is a current challenge in healthcare to detect AD early, when the typical symptoms are minimal. Thus, the search for new AD-specific biomarker molecules has become a highly prioritized task in the AD research community.^2,3^

The main hallmarks of the AD brain are aggregating and accumulating deposits of amyloid-β (Aβ) peptides and hyperphosphorylated tau protein. These deposits are believed to initiate the development of other AD hallmarks: oxidative stress (OS) and chronic inflammation.^4^ OS and inflammation in the brain are associated with disturbed function of neuronal cells, as well as the progressive death of cholinergic neurons in the hippocampus and cortical regions.^4^

OS contributes to AD progression by causing cellular damage. OS development begins when the amount of reactive oxygen species (ROS) increases inside the cell and exceeds the cell's capacity to remove it. ROS are metabolic byproducts, and important regulators of many cellular processes under normal conditions. Antioxidants are protective enzymes in cells, which maintain the balance of ROS and inhibit ROS generation. In many disease states, these defense mechanisms are disrupted, and the amount of ROS exceeds the cell's capacity to remove it. ROS molecules reportedly damage lipids, proteins, and DNA.^4,5^ In particular, ROS are detrimental for mitochondria and endoplasmic reticulum (ER), and dysfunction of these organelles is linked to elevated ROS.^6^ Several alterations in mitochondrial protein functions and structures have been described in the AD brain.^7^ Additionally, the AD brain reportedly exhibits reductions in several protective antioxidative proteins such as superoxide dismutase (SOD), catalase, and peroxiredoxins.^5^ SOD is a mitochondrial enzyme that can neutralize superoxide radicals to protect mitochondria. SOD-positive neurons are specifically localized in the hippocampus and frontotemporal cortex, which are the areas affected in AD.^8^ Proper neuron function requires correct protein synthesis in every step within the ER. Protein synthesis is negatively affected by aging and several neurodegenerative disorders.^9,10^

It has been reported that OS causes protein damage in the AD brain, even in the early stage of disease. Such damage may alter the proteins’ functions and expressions. Tear fluid (TF) is a protein-rich fluid and thus may be an important source of potential AD biomarkers. Moreover, the protein composition of TF is known to reflect aging and pathophysiological changes of AD.^11–13^ A good biomarker molecule is sensitive to both disease type and stage, easy to collect, non-invasive, and cost-effective. TF meets the latter three criteria and is therefore a promising source of biomarkers.

In the present study, we aimed to perform proteomic analysis of TF collected from persons with mild AD dementia and cognitively healthy controls (CO), to compare protein expression levels. We speculated whether upregulated or downregulated protein expression in TF may reflect the underlying AD pathology based on the literature.

## Methods

### Ethics statement

Our study protocol adhered to the principles of the Declaration of Helsinki and was evaluated and approved by the Research Ethics Committee of the Northern Savo Hospital District (Dnro: 482/2017). All study participants were verbally informed, read the information letter, and signed informed consent prior to participation. A proxy consent was signed for individuals diagnosed with dementia-stage AD.

### Study design and protocol

For this cross-sectional pilot study, we recruited a total of 53 volunteers, over 60 years of age, from the Brain Research Unit of the University of Eastern Finland and from the memory clinic of Kuopio University Hospital NeuroCenter. Within this group, 19 volunteers had been diagnosed with AD before enrolling in this study (AD group), and 34 were considered as a cognitively healthy persons, which we used as a control group (CO group). AD was diagnosed by a geriatrician or neurologist at the memory clinics, based on the revised National Institute on Aging and Alzheimer's Association criteria.^14^ Before diagnosing AD, other etiologies of memory decline were excluded by performing brain imaging, comprehensive cognitive testing (Consortium to Establish a Registry for Alzheimer's Disease [CERAD] neuropsychological battery or neuropsychological tests), and differential diagnostic laboratory tests. The exclusion criteria for this study were memory decline due to etiologies other than AD; limited ability to cooperate due to moderate to severe AD; diabetes; and eye diseases, such as glaucoma, dry eye disease, or age-related macular degeneration.

At the first study appointment, comprehensive demographic information was obtained from participants and their family members, including medical history, neurological signs and symptoms, medication, and family history of AD (Table 1). All participants were evaluated using the Clinical Dementia Rating (CDR) scale.^15^ Participants with any signs of upper motor neuron compromise, parkinsonism, balance abnormalities, or ataxia were excluded. All participants were evaluated using the Finnish version of the CERAD neuropsychological test battery, including the Mini-Mental State Examination (MMSE).^16^ Study participants were considered cognitively healthy controls (CO) if their CERAD results were within the normal range, and they experienced no decline in daily activities based on demographic information and CDR interviews (CDR 0).

**Table 1.** Demographic data of the cognitively healthy control (CO) and Alzheimer's disease (AD) study groups.

	CO	AD	*p* *(CO-AD)*
Number of participants, n	34	19	
Age, y (±SD)	71 (5.3)	71 (6.8)	0.817
Gender, female, n (%)	19 (56)	12 (63)	0.413
MMSE result, max 30 (±SD)	28.6 (1.4)	23.9 (2.8)	***0***. ***000***
MMSE, range	25–30	16–27	Nap
Family history, N (%)	19 (57.6)	12 (63.2)	0.462
BMI, kg/m^2^ (±SD)	25.3 (2.6)	24.5 (3.4)	0.338
Coronary artery disease, n (%)	3 (8.8)	2 (10.5)	0.596
Hypercholesterolemia medication, n (%)	14 (41.2)	5 (26.3)	0.218
Hypertension medication, n (%)	19 (55.9)	12 (63.2)	0.413
*APOE* allele 4 carriers, n (%)	15 (44.1)	14 (82.4)	***0***. ***009***

Bold indicates significant differences between groups at *p *≤ 0.05. CO: cognitively healthy control group; AD: Alzheimer's disease group (AD participants represent as a mild AD dementia, CDR 0.5–1.0); SD: standard deviation; MMSE: Mini-Mental State Examination (range 0–30); *APOE*: apolipoprotein E; Nap: not applicable.

### APOE genotyping

Venous blood samples were collected, and genomic DNA was extracted using the QIAamp DNA Blood Mini Kit (QIAGEN). *APOE* alleles were determined using TaqMan genotyping assays (Applied Biosystems [ABI], Foster City, CA, USA) for two single-nucleotide polymorphisms (rs429358 and rs7412), and an allelic discrimination method on the ABI 7000 platform.^17^

### Eye examination

Slit-lamp examination was performed using a CSO biomicroscope (Costruzuzione Strumenti Oftalmici, Firenze, Italy). Tear film stability was assessed based on tear film break-up time (tBUT), and Schirmer test strips were used to measure TF production.^18,19^ Lid margin and bulbar conjunctival redness were scored according to Institute of Eye Research grading scales (very slight, 0; mild, 1; moderate, 2; or severe, 3).^18^ Corneal and conjunctival fluorescein staining patterns were scored using the Oxford grading scale, from 1 (absent) to 5 (marked).^19^

### Tear collection and sample preparation

TF samples were collected using Schirmer strips (Tear Touch; Madhu Instruments) without anesthesia. The strips were placed under the lower eyelid for 5 min. Then the strips were placed in 1.5-ml Eppendorf tubes and stored at −80°C until further processing.

### In-solution protein digestion

Proteins were eluted from the Schirmer strips with 200 µl phosphate-buffered saline and then precipitated with cold acetone. Next, reductive alkylation was performed using DTT and iodoacetamide, followed by in-solution protein digestion with 2 µg trypsin GOLD (Promega, Madison, WI, USA).

### Protein identification and label-free quantification

Tryptic peptides were purified using 10 µl OMIX-C18 micro-SPE pipette tips (Agilent, Santa Clara, CA, USA). Next, the purified peptides were injected into the liquid chromatography/mass spectrometry LC/MS system, comprising a timsTOF Pro (Bruker Daltonik, Bremen, Germany) coupled online to a nanoElute nanoflow liquid chromatography system (Bruker Daltonik, Bremen, Germany) via a CaptiveSpray nanoelectrospray ion source. The LC/MS data were searched against the human Uniprot database (20,431 entries), using PEAKS X + software version 10.5 (Bioinformatics Solutions, Waterloo, ON, Canada). A false-discovery rate (FDR) of 1% was applied to the data sets.

For label-free quantification using PEAKS, we performed ID-directed label-free quantification with outlier removal. The following parameters were applied regarding peptide features: quality ≥ 4, peptide ID count per group ≥ 1, and detected in ≥ 1 sample per group. The following parameters were applied to the proteins: FDR ≤ 5%, fold change ≥ 2, and significance on analysis of variance with ≥ 2 peptides. The significance score is calculated as the −10log10 of the significance testing p-value.

### Statistical analysis

Statistical analyses were performed using SPSS 22 software (SPSS Inc., Chicago, IL, USA). A *p* value of <0.05 indicated a significant finding. Results regarding demographic data, CERAD test (MMSE), and ocular data (Table 2) are presented as mean ± standard deviation (SD), or as number of cases and proportions (%). We performed t-test and one-way analysis of variance, followed by a least-significant difference post-hoc test. Group differences were expressed as mean difference and 95% confidence intervals.

**Table 2.** Ocular data for control (CO) and Alzheimer's disease (AD) study groups.

Ocular data	CO *n = 34*	AD *n = 19*	*p* *(CO-AD)*	*Mean difference* *(95% CI)*
Schirmer, mm in 5 min, right eye	9.28 (6.9)	8.3 (8.2)	*0*.*648*	*0.96* *(−3.53–5.45)*
Schirmer, mm in 5 min, left eye	9.03 (6.1)	11.2 (8.3)	*0*.*281*	*−2,13* *(−6.52–2.26)*
TBUT, right eye	5.54 (3.1)	5 (3.8)	*0*.*579*	*0.54* *(−1.56–2.64)*
TBUT, left eye	5.6 (3.2)	5 (3.8)	*0*.*545*	*0.6* *(−1.51–2.71)*
Ocular data		CO *n = 34*	AD *n = 19*	*p* *(CO-AD)*
Conjunctival redness, right eye				*0*.*025*
0 = no redness, % (n)		0	0	
1 = mild redness, % (n)		58.3 (21)	85 (17)	** **
2 = moderate redness, % (n)		41.7 (15)	10.0 (2)	
3 = severe redness, % (n)		0	5 (1)	
Conjunctival redness, left eye				*0*.*062*
0 = no redness, % (n)		0	0	
1 = mild redness, % (n)		58.3 (21)	80.0 (16)	
2 = moderate redness, % (n)		41.7 (15)	15.0 (3)	
3 = severe redness, % (n)		0	5 (1)	
Lid margin redness, right eye				*0*.*162*
0 = no redness, % (n)		0	0	
1 = mild redness, % (n)		69.4 (25)	45.0 (9)	
2 = moderate redness, % (n)		25.0 (9)	50.0 (10)	
3 = severe redness, % (n)		5.6 (2)	5.0 (1)	
Lid margin redness, left eye				*0*.*266*
0 = no redness, % (n)		2.8 (1)	0	
1 = mild redness, % (n)		66.7 (24)	45.0 (9)	
2 = moderate redness, % (n)		25.0 (9)	50 (10)	
3 = severe redness, % (n)		5.6 (2)	5 (1)	
Conjunctival fluorescein staining, right eye				*0*.*108*
0 = absent/very slight, % (n)		5.6 (2)	25 (5)	
1 = mild, % (n)		80.6 (29)	65 (13)	
2 = moderate, % (n)		13.9 (5)	10 (2)	
Conjunctival fluorescein staining, left eye				*0*.*207*
0 = absent/very slight, % (n)		5.6 (2)	20 (4)	
1 = mild, % (n)		77.8 (28)	60 (12)	
2 = moderate, % (n)		16.7 (6)	20 (4)	
Cornea fluorescein staining, right eye				*0*.*503*
0 = absent/very slight, % (n)		44.4 (16)	60 (12)	
1 = mild, % (n)		38.9 (14)	25 (5)	
2 = moderate, % (n)		16.7 (6)	15 (3)	
Cornea fluorescein staining, left eye				*0*.*656*
0 = absent/very slight, % (n)		47.2 (17)	60 (12)	
1 = mild, % (n)		33.3 (12)	25 (5)	
2 = moderate, % (n)		19.4 (7)	15 (3)	

Results presented as measurement results (SD), or number (percentage). Bold indicates significant differences between groups at *p *≤ 0.05. SD: standard deviation; n: number; CO: healthy control group; AD: Alzheimer's disease group (AD participants represent as a mild AD dementia, CDR 0.5–1.0); TBUT: tear breakup time; 95% CI: 95% confidence interval.

## Results

### Demographic data

Table 1 presents the participants’ demographic data. The two groups significantly differed in MMSE results, and proportion of *APOE* allele 4 carriers. Mean MMSE scores were 28.6 (±1.4) in the CO group, and 23.9 (±2.8) in the AD group (*p *< 0.000). The proportion of *APOE* allele 4 carriers was 44.1% in the CO group, and 82.4% in the AD group (*p *< 0.009).

### Ocular data

TF production (Schirmer test) was measured in both eyes. The two study groups did not significantly differ in TF production, TF break-up time (TBUT), lid margin redness, conjunctival redness, or ocular surface staining (OSS) (Table 2).

### Proteomics data

A total of 47 proteins were identified in TF samples. We categorized these proteins based on their function. This study we report 16 proteins that were upregulated by over two-fold in the AD study group compared to the CO group, and that had an FDR of ≤5% (Table 3). We searched the literature for information regarding the roles of these proteins in cells, and how the protein dysfunction may relate to AD or the process of dementia.

**Table 3.** Upregulated and downregulated tear fluid (TF) proteins in participants with mild Alzheimer's disease (AD) dementia compared with cognitively healthy controls (CO), given as ratio of CO to AD values.

Protein name	Symbol	Ratio CO:AD	*Significance score*
***Upregulated***			
Nucleosome assembly protein 1-like 4	NP1L4	**6**.**37**	38
Gamma butyrobetaine dioxygenase	BBOX1	**6**.**37**	59
Cystatin C	CYTC	**4**.**36**	98
Ribonuclease 4	RNAS4	**3**.**86**	23
Pterin-4-alpha-carbinolamine dehydratase	PCD	**3**.**19**	88
Ribonuclease T2	RNT2	**2**.**87**	24
Aldehyde dehydrogenase family 1 member A3	AL1A3	**2**.**52**	46
Serine-tRNA ligase cytoplasmic	SYSC	**2**.**48**	38
Triosephosphate isomerase	TPIS	**2**.**3**	77
Clathrin heavy chain 1	CLH1	**2**.**21**	24
Phosphoglycerate mutase 1	PGAM1	**2**.**16**	54
Eukaryotic translation initiation factor 3 subunit L	EIF3L	**2**.**1**	60
Cytosolic purine 5'nucleotidase	5NTC	**2**.**09**	50
Heterogenous nuclear ribonucleoprotein A2/B1	HNRNPA2B1	**2**.**04**	84
Glycogen phosphorylase liver form	PYGL	**2**.**03**	95
ERO1-like protein alpha	ERO1α	**2**.**01**	69
***Downregulated***			
Peroxiredoxin-5 mitochondrial	PRDX5	0.61	not sig.

The differences between AD and CO were considered significant (*p *< 0.05) if the ratio value was <0.5 or >2.0. Significant values are marked in **bold**. ANOVA was applied as significance method with at least two used peptides. The significance score is calculated as the −10log10 of the significance testing p-value.

The following proteins were significantly upregulated in the AD group, ordered according to the observed fold change: nucleosome assembly protein 1-like 4 (NP1L4), gamma butyrobetaine dioxygenase (BBOX1), cystatin C (CYTC), ribonuclease 4 (RNAS4), pterin-4-alpha-carbinolamine (PCD), ribonuclease T2 (RNT2), aldehyde dehydrogenase family 1 member A3 (AL1A3), serine-tRNA ligase cytoplasmic (SYSC), triosephosphate isomerase (TPIS), clathrin heavy chain 1 (CLH1), phosphoglycerate mutase 1 (PGAM1), eukaryotic translation initiation factor 3 subunit L (ElF3L), cytosolic purine 5ʹ nucleotidase (5NTC), heterogenous nuclear ribonucleoprotein A2/B1 (HNRNPA2B1), glycogen phosphorylase liver form (PYGL), and ERO1-like protein alpha (ERO1α).

## Discussion

Proteins in TF reflect pathophysiological changes in many systemic diseases, including AD, and thus TF is a potential source of biomarker candidates to detect early AD.^11,13^ In this pilot study, we analyzed TF proteins from both individuals with mild AD and cognitively healthy controls (CO). Totally we founded 47 altered proteins from AD group which categorized based on their functions. Here, we reported 16 altered TF proteins NP1L4, BBOX1, CYTC, RNAS4, PCD, RNT2, AL1A3, SYSC, TPIS, CLH1, PGAM1, EIF3L, 5NTC, HNRNPA2B1, PYGL, and ERO1α that were significantly upregulated in AD group. Here we discuss whether these proteins’ function link to AD pathology and whether these proteins are potential biomarker candidate.

It was important to eliminate factors that are not related to AD and that may affect the level of TF proteins. Therefore, we ensured that the CO and AD study groups were homogenous in their demographics, eye data, and TF production. We found no difference in TF production between the CO and AD study groups, which is line with previous findings.^13^ Participants with eye disorders and diabetes were excluded from both groups. The AD and CO groups differed only in their MMSE results (worse in the AD group), and proportion of *APOE4* carriers (higher in AD group). All AD participants represent as a mild stage of disease (CDR 0.5–1.0).

OS develops in the brain when the protection against ROS is disrupted, as has been shown to occur in AD.^4^ Aldehyde dehydrogenases (ALDHs) are enzymes that metabolize both endogenous and exogenous reactive compounds, regulate several cell functions, and participate in the cellular response to OS. The roles of ALDHs have been described as both beneficial and harmful.^20^ In our study, the AD study group exhibited upregulated expression of aldehyde dehydrogenases family 1 member A3 (AL1A3), which is reportedly expressed in cornea, and may have important roles in the protection of epithelial cells against OS.^21^ AL1A3 has also been described as having chaperone-like activities against protein aggregation in epithelial cells.^22,23^ In AD, aggregating proteins Aß peptide and tau are detected in the eyes,^24^ indicating the importance of chaperone-like activities in AD. Upregulated AL1A3 may indicate the presence of both OS- and AD-linked proteins in the eyes of persons with mild AD dementia.

The AD study group also exhibited upregulation of Pterin-4-alpha-carbinolamine dehydratase (PCD), which is an enzyme involved in the biosynthesis of tetrahydrobiopterin (BH4). PCD is an important cofactor in the synthesis of several neurotransmitters, including dopamine, serotonin, norepinephrine, epinephrine, and nitric oxide. BH4 also reportedly shows anti-inflammatory and antioxidant properties against free radicals.^25,26^ BH4 and its metabolites are decreased in AD, with the exception of one BH4 derivative, neopterin. Neopterin is upregulated in inflammatory processes and is detected at increased levels in the plasma and cerebrospinal fluid of persons with AD.^26^ Since, we did not detect BH4 or neopterin in the TF samples from our AD study group, we anticipate that PCD upregulation might be a response to increased OS.

In the AD group, we also detected upregulation of the following six proteins that are involved in protein synthesis: EIF3L, ERO1-like protein alpha (ERO1α), HNRNPA2B1, serine-tRNA ligase cytoplasmic (SYSC), ribonuclease T2 (RNT2), and ribonuclease 4 (RNAS4). EIF3 is the key regulator of translation initiation, and is a large protein complex comprising 13 subunits, including EIF3L.^27^ The functions of the EIF3 complex are reportedly important for neurons during dendritic pruning. Altered expression of the EIF3 complex is correlated with Parkinson's disease progression.^28^ Our present findings suggest that inappropriate levels of EIF3 s may also interfere with the initiation of protein translation in neurons and may also occur in AD. Furthermore, the function of the EIF3L subunit has been linked to aging and ER stress. A loss-of-function study in *C. elegans* revealed that EIF3L gene mutation increases lifespan and decreases ER stress.^27^ Our present finding of upregulated EIF3L expression in the AD group may indicate increased ER stress in mild AD dementia.

ERO1α is a highly conserved ER protein that is responsible for correct folding of oxidative proteins. Together with disulfide isomerase (PDI), ERO1α catalyzes the formation of disulfide bonds in the ER. Dysregulation of these processes leads to cell and tissue damage and is associated to several neurodegenerative diseases.^29^ ERO1α and PDI are both reportedly upregulated in several cell types under pathological conditions.^29^ In line with a previous study, we detected ERO1α upregulation in the AD study group.

HNRNPA2B1 is nuclear RNA-binding protein, and an important regulator of RNA metabolism, trafficking, miRNA export, and gene expression. The function of HNRNPA2B1 is regulated by cholinergic signaling. The AD brain suffers the loss of cholinergic neurons, leading to long-term cholinergic deficiency. In a murine model of AD, reduced cholinergic signaling negatively affects the transcriptional process mediated by HNRNPA2B1.^30,31^ In our present study, HNRNPA2B1 was upregulated in the AD group, which seems to be in contrast to the observations in AD model mice.^30,31^ This difference may be explained by the fact that the participants in our study had mild AD dementia, and thus may have had less robust cholinergic deficiency compared to AD mice.

RNT2 and RNAS4 are RNA-binding proteins, and both act as crucial regulators of protein synthesis by controlling RNA longevity.^32,33^ Unsurprisingly, dysregulation of RNA-binding proteins has negative effects on cellular homeostasis, which are linked with many disorders.^32,33^

RNT2 is ubiquitously expressed, and it is associated with immune system regulation, angiogenesis repression, cell differentiation, and apoptosis.^34,35^ Notably, increased ROS reportedly induces RNT2 expression, and RNT2 upregulation is associated with increased ROS-induced cell death and increased antioxidant capacity of RNT2.^35^ However, the exact role of RNT2 enzyme upregulation during AD progression is poorly understood. Our present results support the earlier finding that RNT2 upregulation may indicate increased ROS production in AD study group.^35^

RNAS4s are found in mucosal secretions, and their physiological role is largely unknown. RNAS4 is speculated to be involved in the defense system and is reportedly overexpressed in microglia among patients with chronic pain.^36^ It appears that RNAS4 regulates neurons and synaptic plasticity via microglia and by secreting cytokines and interleukins.^37^ In amyotrophic lateral sclerosis (ALS), RNAS4 is co-expressed with angiogenic ribonuclease, which may indicate that RNAS4 has angiogenic, neurogenic, and neuroprotective functions. Additionally, RNAS4 was found to be decreased in ALS, and administration of RNAS4 was beneficial for motor neuron survival in a mouse model.^32^ The literature does not yet include information regarding the role of RNAS4 in AD. It can be speculated that RNAS4 upregulation is a response to the harmful brain milieu, and may indicate that neuronal loss has occurred, and the need for neuroprotection in mild AD dementia.

We also detected upregulated SYSC in the AD study group. SYSC is a ligase that reportedly regulates vascular endothelial growth factor A (VEGF-A) during hypoxia.^38^ VEGF-A overexpression is associated with vascular abnormalities and may contribute to various diseases.^39^ In AD, VEGF-A upregulation is also associated with cerebrovascular deficits. Furthermore, VEGF-A reportedly binds Aβ peptide and co-localizes with amyloid plaques in the AD brain, and VEGF-A expression is thought to positively correlates with AD severity.^39^ Additionally, VEGF-A has been shown to be protective against AD-related neurotoxicity.^40^ Our present results may indicate that vascular deficits and AD-related protein already present in the brain during mild AD dementia.

Our AD study group also exhibited upregulation of three cytoplasmic proteins: NP1L, cystatin C (CYTC), and clathrin heavy chain 1 (CLH1). Nucleosome assembly proteins belong to a family of shuttle proteins, which regulate lipid-mediated signal transduction via diacylglycerol kinases (DGK). Under pathological conditions, NAP1Ls can rapidly respond and translocate DGK from the nucleus to the cytoplasm. Additionally, NAP1L is suggested to be an anchoring protein for DGKs, and it has been speculated that increased NP1L levels are protective against the cytotoxic effects.^41^ In our study, NP1L4 expression was over 6-fold higher in the AD study group than the CO group, and this high upregulation may indicate the importance of this protein. High NP1L4 levels likely indicate increased cytotoxicity in the brain milieu. Intriguingly, in our study, the study participants with mild AD dementia already had high levels of NP1L4, suggesting that NP1L4 may be a potential AD biomarker candidate.

CYTC is cysteine protease inhibitor expressed in all cells and secreted in many body fluids. CYTC activity increases during the inflammation response and OS,^42^ and increased CYTC levels have been reported in AD.^43^ We detected upregulated CYTC in the AD study group. It is possible that CYTC plays a protective role during AD progression, and that high CYTC plasma levels may indicate poor cognitive performances.^43^ CYTC is an inhibitor of cathepsins, which are important cellular proteases. Increased cathepsin D levels have been linked with AD.^44^ Moreover, CYTC reportedly colocalizes with Aβ peptide in the AD brain,^45^ and inhibits the enzymes involved in the degradation of Aβ peptide into toxic Aβ aggregates.^46^ Genetic analyses have identified the CYTC haplotype CST3 B as a risk factor for AD.^42^ The role of CYTC in the AD brain is well-described in the literature, and it appears that the altered CYTC plays a role in AD progression. Our present results are in line with earlier findings,^43^ and provide additional evidence that CYTC levels are higher in AD. This suggests that CYTC may serve as a useful AD biomarker protein.

CLH1 is the main protein component of the coated vesicles of cytoplasmic organelles.^47^ Neurons exhibit particularly high CLH1 content, especially at the synaptic terminal, and varying according to the neuronal activity.^47^ Communication between neurons is mediated by recycled coated vesicles, in a process termed clathrin-mediated endocytosis. Thus, CLH1 seems to be an important factor for synaptic transmission.^47^ Here we found that CLH1 was upregulated in the AD study group, indicating increased clathrin-mediated endocytosis in AD. These results support the earlier findings of increased endocytosis in persons with AD.^48^

In our AD group, we also found four upregulated proteins that can be linked to regulation of energy metabolism^49–52^; gamma BBOX1, PGAM1, TPIS, and glycogen phosphorylase liver form (PYGL). Glucose metabolism and ATP production are essential for energy production in the brain, and for maintaining normal synaptic functions. Brain energy production is reportedly disrupted in many ways in the AD brain.^49–52^

Finally, our AD study participants exhibited overexpression of cytosolic purine 5ʹ nucleotidase (5NTC), which is linked with cellular purine metabolism. This enzyme regulates cell maintenance, proliferation, migration, and differentiation.^53^ Notably, 5NTC is also associated with brain arterial diameters (BAD) genes. Intracranial arterial diameter may be a predictor of brain health and aging, because very small and very dilated arterial diameters are both associated with higher risk of vascular events, and arterial dilation is associated with dementia and cognitive decline.^54^ The 5NTC upregulation observed in our study may indicate dilated arteria in our AD study participants.

Overall, our present results support the hypothesis that TF is a potential source of AD biomarkers, and that protein expression levels in TF may reflect underlying pathophysiological processes of AD or the continuum of dementia. Although this was a pilot study with a relatively small sample size, we identified 16 potential biomarker candidate proteins, which have functions linked to increased OS, protein synthesis, and disturbed energy metabolism and, thus may be linked to pathophysiological changes examined in AD. According to the previous studies, CYTC has been identified as a potential biomarker candidate for AD and our results supported these results. For other proteins, further studies are needed to demonstrate whether the protein alterations detected in this study can be linked to pathophysiological changes occurring in degenerative processes in brain or not.

These findings may lead to novel methods for recognizing AD earlier, based on TF analysis, and encourage us to continue this line of investigation with a larger study cohort and new research questions. Moreover, this study may provide new information about the AD-related pathophysiological changes at the cellular level and can be useful for research purposes beyond searching for novel biomarkers.

### Conclusion

We conclude that this study provides us a new information about the TF proteins which may reflect underlying pathophysiological changes in AD or dementia. This study encourages us to continue further examine to develop new easy-to-collect and non-invasive TF biomarkers for recognize early AD.
